# Catheter Ablation of Ischemic Ventricular Tachycardia Originating from an Inferobasal Right Ventricular Scar Using Substrate Mapping: A Case Report

**DOI:** 10.19102/icrm.2018.090604

**Published:** 2018-06-15

**Authors:** Daniel A. Sohinki, Hiroshi Nakagawa, Stavros Stavrakis

**Affiliations:** ^1^Department of Cardiology, University of Oklahoma Health Sciences Center, Oklahoma City, OK, USA; ^2^Heart Rhythm Institute, University of Oklahoma Health Sciences Center, Oklahoma City, OK, USA

**Keywords:** Catheter ablation, ischemic heart disease, ventricular tachycardia

## Abstract

Catheter ablation of ventricular tachycardia (VT) has emerged as a superior alternative to antiarrhythmic drug therapy in patients with ischemic cardiomyopathy, with the vast majority of ischemic VT being ablation from the endocardial surface of the left ventricle (LV). While rare, the possibility of ischemic right ventricular (RV) VT should also be entertained, especially in patients with previous myocardial infarction and in those individuals in whom LV endocardial ablation fails to abolish VT. Further, success rates remain disappointing in some of these cases, often owing to difficulties in mapping the tachycardia due to hemodynamic instability during VT. We report a case of hemodynamically unstable ischemic VT successfully ablated from the endocardial surface of the LV and RV using a substrate mapping approach in a patient with a large inferior myocardial infarction, involving RV infarction.

## Introduction

Radiofrequency (RF) catheter ablation has been shown to be effective in patients with recurrent ventricular tachycardia (VT) and has been indicated as superior to escalating antiarrhythmic drug (AAD) therapy in patients with ischemic cardiomyopathy (ICM).^1^ However, success rates have been reported to vary between 50% to 76% in patients with structural heart disease, owing to VT non-inducibility, difficulties in mapping, and hemodynamic instability during VT.^2,3^ While the majority of ischemic VTs are ablated from the endocardial surface of the left ventricle (LV),^4^ failure to abolish VT after ablation at these sites should prompt a search for circuits in other areas of the heart, including via a pericardial approach and at the right ventricular (RV) endocardium. We report a case of scar-related VT successfully ablated from the endocardial surface of the inferobasal RV and LV in a patient with ICM.

## Case presentation

A 58-year-old male with a past history of ICM and LV systolic dysfunction (LV ejection fraction of 30%) was referred to our electrophysiology laboratory for RF catheter ablation of VT. He had a previously placed, primary-prevention dual-chamber implantable cardioverter-defibrillator (ICD) and a recent history of multiple appropriate shocks for VT that had been recurrent despite escalating doses of amiodarone to 400 mg daily and metoprolol succinate to 150 mg daily, along with the addition of mexiletine 150 mg three times daily. Stress myocardial perfusion imaging demonstrated scarring in the inferior, inferolateral, and apical LV walls but did not reveal evidence of reversible ischemia. Data from transthoracic echocardiography (TTE) were concordant, showing akinesis and aneurysmal deformity of the inferior and inferolateral LV myocardium. The RV was poorly visualized on TTE, though on intracardiac echocardiography (ICE), the inferior and inferobasal RV were hypokinetic, with a thin, hyperechoic appearance of the myocardium.

After verbal and written informed consent were obtained, the patient was brought to the electrophysiology laboratory for the procedure and placed under general anesthesia. Venous access was obtained and diagnostic catheters were placed in the RA appendage, His position, and RV. An ICE catheter was used to guide transseptal catheterization and for image integration using the CartoSOUND™ enhanced mapping software (Biosense Webster, Diamond Bar, CA, USA). Three-dimensional electroanatomic mapping of the LV and RV was performed using the CARTO™ 3 RMT mapping system (Biosense Webster, Diamond Bar, CA, USA) and a magnetic catheter maneuvering system (Epoch^®^; Stereotaxis, Inc., St. Louis, MO, USA).

**Figure 1:**
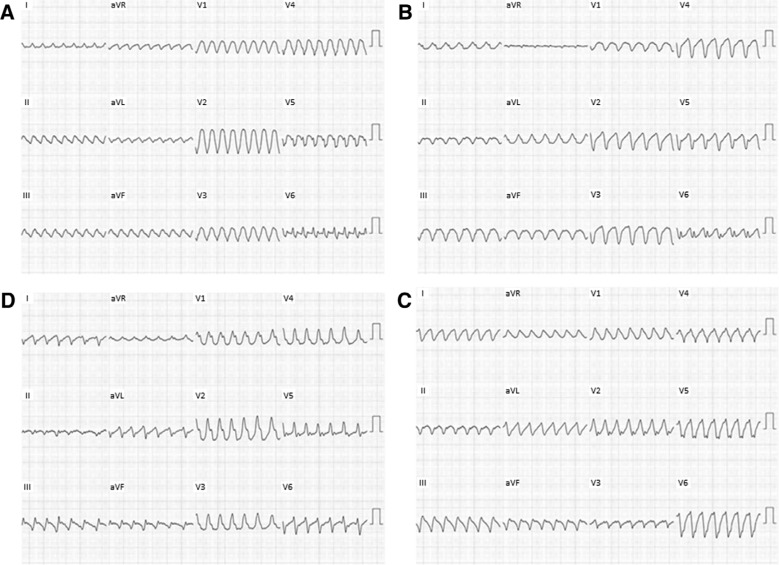
VT morphologies induced during electrophysiology study. **A:** VT-1 had LBBB morphology with superior axis. **B:** VT-2 had LBBB morphology with superior axis. **C:** VT-3 had right bundle branch block morphology with superior axis. **D:** VT-4 had right bundle branch block morphology with superior axis.

**Figure 2:**
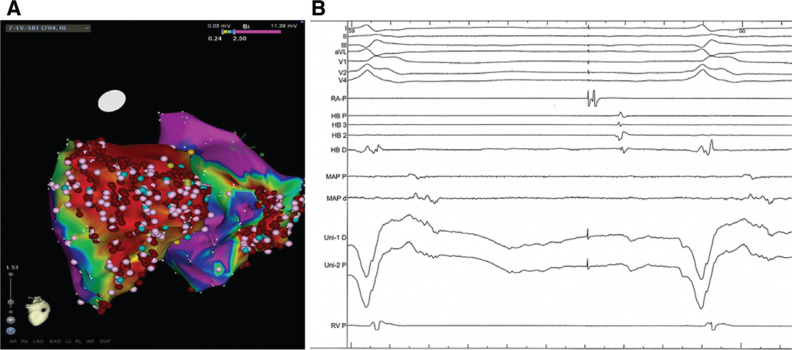
LV voltage mapping. **A:** Bipolar voltage map of the inferior LV wall. The pink tags correspond to late potentials and fractionated electrograms. The red tags correspond to ablation sites. The voltage range is 0.24 mV to 2.5 mV. **B:** Late fractionated potentials recorded from the inferior LV base. MAP and Uni are bipolar and unipolar electrograms recorded from the mapping catheter, respectively.

**Figure 3:**
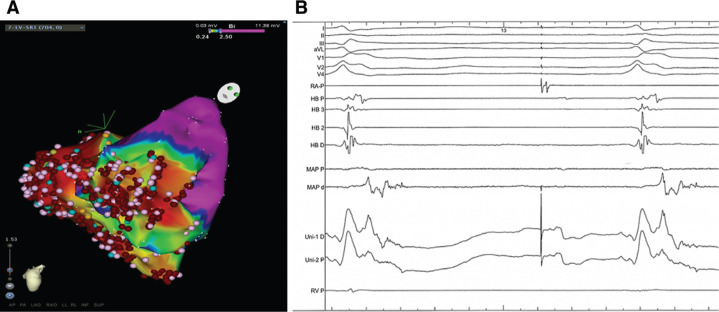
RV voltage mapping. **A:** Bipolar voltage map of the RV viewed from a right anterior oblique caudal projection. The pink tags correspond to late potentials and fractionated electrograms. The red tags correspond to ablation sites. The voltage range is 0.24 mV to 2.5 mV. **B:** Late fractionated potentials recorded from the RV inferior base near the tricuspid annulus. MAP and Uni are bipolar and unipolar electrograms recorded from the mapping catheter, respectively.

At the beginning of the study, four VT morphologies were easily inducible using burst pacing from the RV **(Figure 1)**. VT-1 and VT-2 both had left bundle branch block (LBBB) morphology with a superior axis. VT-3 and VT-4 had a right bundle branch block morphology, again with superior axis. All four VTs were hemodynamically unstable and required direct-current cardioversion. Because of this, we opted to perform substrate mapping during RA appendage pacing (pacing cycle length: 800 ms) to identify isolated late ventricular potentials (ILPs), as previously described.^5^ We first performed mapping of the LV, identifying late fractionated ILPs in the inferobasal, inferolateral, and apical walls **(Figure 2)**. A total of 42 RF applications (RF power: 30–50 watts) were delivered in the inferior RV endocardium, targeting ILPs. However, after LV endocardial ablation was complete, VT-2 (LBBB and superior axis morphology) was still easily inducible. We therefore decided to map the RV, again targeting ILPs. A large area of low bipolar voltage (< 0.5 mV) was identified in the inferobasal RV wall with associated ILPs **(Figure 3)**. Pacemapping from this area showed a similar morphology to those of VT-1 and VT-2. A total of 38 RF applications were delivered in the inferior RV endocardium, targeting ILPs within the low voltage area. Following ablation of ILPs in the RV, VT was no longer inducible and the study was completed.

The patient remained free of ICD discharges during hospitalization and was continued on AAD therapy with amiodarone, mexiletine, and metoprolol succinate on discharge. The patient is still alive and has not received any shocks four months after ablation.

## Discussion

This case highlights the potential importance of RV VT in patients with ischemic cardiomyopathy and shows that a substrate-mapping approach can successfully guide ablation in these cases. Catheter ablation of RV VT is typically discussed in the setting of RV outflow tract VT; RV scarring related to surgery for congenital heart disease (CHD); infiltrative cardiomyopathies; scarring in patients with arrhythmogenic right ventricular cardiomyopathy (ARVC); or, rarely, in patients with Brugada syndrome.^6–8^ We report a case of successful RF catheter ablation of VT originating from an inferobasal RV scar in a patient with ICM. While rare in isolation, RV myocardial infarction (MI) complicates 25% to 50% of inferior MIs^7,9,10^ and is therefore capable of producing the substrate for reentrant VT. However, the occurrence of ischemic RV VT is confined to case reports^11,12^ and optimal mapping and ablation techniques have not been described. Of note, the clinical presentations reported in cases of ischemic RV VT vary significantly. Menz et al. describe two patients who underwent successful VT ablation from the RV septum after failed ablation in the LV septum, though one presented with a prior inferior infarct and the other presented with a prior anterior infarct.^12^ A third case described by Shimizu et al. involved successful VT ablation from the RV free wall of a patient with a prior RV infarct.^11^ All VT in these cases had LBBB morphology.

Despite the rarity of ischemic RV VT, approximately one-third of ischemic VT originating from the LV demonstrated a LBBB morphology on the electrocardiogram (ECG),^10^ which might suggest an RV origin and thus cause confusion regarding VT localization. Several factors limit the usefulness of the surface ECG in this regard, including the presence and degree of prior myocardial scar, a lack of concordance between the scar and exit site locations, heart morphology and variations in chest wall anatomy, the effects of concomitant medical therapy, and the integrity of the His-Purkinje system.^13^ Nonetheless, the surface ECG can provide the electrophysiologist with clues regarding the “regionalization” of the VT, as a first step towards successful mapping and ablation. As alluded to, a QS pattern in lead V1 (ie, a LBBB morphology) is suggestive of an RV VT origin because the LV is activated late via slow myocardial conduction, similar to in the case of true LBBB. While VT originating in the LV septum can have a similar appearance, septal VT tends to have a narrower QRS as compared with VT originating in the free wall, though anisotropic myocardial conduction (as may present in patients with other areas of scar of infiltrative disease) may invalidate this assumption.^13^ In this case, the VT-2 morphology, which predominated after LV substrate modification, was very wide and would be less likely to be present originating from the LV septum as based on the surface ECG. However, other generalizations regarding VT origin based on surface ECG morphology may not hold true. For example, it has been reported that negative precordial concordance is suggestive of an apical sepal origin,^13^ yet, in this case, VT-2 showed negative concordance and was successfully ablated from the inferobasal RV. These contradictions may be related to different exit sites from the scar into the myocardium, presence of other abnormal areas of myocardium, or incorrect assumptions regarding the position of the heart in the chest, as previously mentioned. Because of these issues, the possibility of an RV origin of the VT should be thoroughly examined irrespective of QRS morphology on the surface ECG, whenever ablation in the LV fails to eliminate the VT.

In our case, extensive LV endocardial ablation of ILPs within the areas of low bipolar voltage (< 0.5 mV) failed to abolish VTs with LBBB morphologies, which prompted a search for an alternative endocardial origin of the arrhythmia. While most VT in patients with ICM is felt to originate from the LV subendocardium, the importance of alternative sites in patients with ischemic heart disease (eg, epicardial, RV subendocardial) should not be overlooked, especially in cases in which VT remains inducible following extensive LV ablation, or when sites of earliest activation are found in the LV septum.^12^ In a case series of 18 patients who presented for VT ablation with evidence of prior septal infarction, 11 (61%) had evidence of RV scarring as assessed by bipolar voltage mapping, 73% of whom were confined to the RV septum. Additionally, 10% of the inducible VTs had a critical isthmus in the RV septum and 82% of these could only be successfully ablated from the RV. All of the RV VTs in this series had LBBB morphology.^14^ Our case demonstrates that, in addition to the interventricular septum, the inferobasal RV may also be a site of origin for ischemic VT, especially in patients with prior inferior MI.

While mapping during VT is ideal in order to fully define the reentrant circuit and identify earliest activation, this is often not possible due to hemodynamic instability, as occurred in this case. Substrate mapping has emerged as an alternative to activation mapping in patients with hemodynamically unstable or otherwise unmappable VT.^5,15^ Recent data suggest that VT induction and mapping before ablation prolongs procedure times, increases radiation exposure, and increases the need for electrical cardioversion without improving ablation efficacy,^5^ thus potentially favoring a substrate mapping approach in some patients with scar-related VT. The approach involves identifying areas of low voltage and corresponding ILPs that represent surviving myocardial bundles within the infarcted scar. In general, normal tissue voltage is defined as > 1.5 mV, where areas of myocardium with < 0.5 mV are felt to represent scar, though these definitions vary based on endocardial, mid-wall, and epicardial location of scar as well as the underlying disease process.^5^ Studies comparing VT ablation using induction and activation mapping versus substrate mapping have yielded conflicting results, though these studies vary with their respect to timing of late potentials, scar voltage, degree of fractionation, and etiology of VT.^5,15^ In this case, we were able to identify ILPs in the inferobasal RV that corresponded to an area of low bipolar voltage (< 0.5 mV). Ablation in this area rendered the patient’s VT noninducible, despite being unable to identify sites of early activation during VT.

## Conclusion

This case demonstrates the potential importance of RV endocardial sites of VT origin in patients with ICM, especially those with previous inferior MI involving RV infarction. Mapping in these areas should be considered in patients whose VT remains inducible despite ablation in the LV endocardium, or in patients in whom earliest activation during VT is recorded from sites in the LV septum. Further, this case demonstrates the feasibility of substrate mapping to guide ablation in the RV in patients with ischemic RV scarring. Finally, ECG criteria for differentiating the RV from the LV for origin of VT are imperfect, and it is important to recognize the possibility of an RV origin, irrespective of QRS morphology, on the surface ECG in patients with ICM.
